# Lithium Decreases Glial Fibrillary Acidic Protein in a Mouse Model of Alexander Disease

**DOI:** 10.1371/journal.pone.0138132

**Published:** 2015-09-17

**Authors:** Christine M. LaPash Daniels, Elizabeth Paffenroth, Elizabeth V. Austin, Konstantin Glebov, Diana Lewis, Jochen Walter, Albee Messing

**Affiliations:** 1 Waisman Center, University of Wisconsin-Madison, Madison, Wisconsin, United States of America; 2 Department of Comparative Biosciences, University of Wisconsin-Madison, Madison, Wisconsin, United States of America; 3 Department of Neurology, University of Bonn, Bonn, Germany; University of Florida, UNITED STATES

## Abstract

Alexander disease is a fatal neurodegenerative disease caused by mutations in the astrocyte intermediate filament glial fibrillary acidic protein (GFAP). The disease is characterized by elevated levels of GFAP and the formation of protein aggregates, known as Rosenthal fibers, within astrocytes. Lithium has previously been shown to decrease protein aggregates by increasing the autophagy pathway for protein degradation. In addition, lithium has also been reported to decrease activation of the transcription factor STAT3, which is a regulator of GFAP transcription and astrogliogenesis. Here we tested whether lithium treatment would decrease levels of GFAP in a mouse model of Alexander disease. Mice with the *Gfap*-R236H point mutation were fed lithium food pellets for 4 to 8 weeks. Four weeks of treatment with LiCl at 0.5% in food pellets decreased GFAP protein and transcripts in several brain regions, although with mild side effects and some mortality. Extending the duration of treatment to 8 weeks resulted in higher mortality, and again with a decrease in GFAP in the surviving animals. Indicators of autophagy, such as LC3, were not increased, suggesting that lithium may decrease levels of GFAP through other pathways. Lithium reduced the levels of phosphorylated STAT3, suggesting this as one pathway mediating the effects on GFAP. In conclusion, lithium has the potential to decrease GFAP levels in Alexander disease, but with a narrow therapeutic window separating efficacy and toxicity.

## Introduction

Alexander disease is a rare and fatal neurodegenerative disease characterized by the formation of protein inclusions, called Rosenthal fibers, in astrocyte cell bodies and processes. Nearly all cases are associated with dominant mutations in the intermediate filament glial fibrillary acidic protein (GFAP) [1], which appear to act in a gain-of-function fashion. Onset can occur throughout the lifespan, with symptoms such as seizures and psychomotor developmental delays common in the younger patients and difficulties with speech and swallowing, autonomic dysfunction, and gait disturbances more common in the older patients [2]. Alexander disease is classified among the leukodystrophies because of the prominent defects in white matter that are present, particularly in early-onset patients.

While the question of whether Rosenthal fibers themselves are toxic is unresolved, a leading hypothesis to explain pathogenesis is that GFAP protein levels accumulate above a toxic threshold (as yet undefined), leading to astrocyte dysfunction and the plethora of secondary effects on the other cell types of the central nervous system [3]. Indeed, GFAP increases are consistently found in Alexander disease, both when measured in brain parenchyma as well as in CSF [4, 5]. GFAP is a major component of the Rosenthal fibers, and in fact the formation of Rosenthal fibers can be initiated simply by over-expressing even wild-type GFAP to high enough levels [6]. The accumulation of GFAP that is found in Alexander disease results in part from increased synthesis, as mRNA levels are increased [7] and there is a spontaneous increase in the activity of the GFAP promoter [8]. In addition, complex changes occur in the degradation pathways. For instance, Alexander disease mutant GFAP activates a c-Jun N-terminal kinase (JNK) stress response that blocks proteasome activity [9, 10]. Together, the alterations in synthesis and degradation could lead to positive feedback loops that exacerbate GFAP accumulation.

Reducing the GFAP accumulation below toxic levels has been proposed as one potential strategy for treatment [11]. Considering degradation pathways as a therapeutic target, one possibility to reduce protein aggregation is the induction of autophagy. Autophagy (macro-autophagy) is a non-specific degradative pathway for long-lived cytoplasmic proteins, protein complexes, and organelles [12]. Autophagy-inducing drugs have proved useful for decreasing protein aggregates in models of other neurodegenerative disorders, such as Huntington’s disease, Parkinson’s disease, spinocerebellar ataxia, and tauopathy [13–17]. Indeed, based largely on cell culture models, autophagy appears to be naturally increased in Alexander disease patients, and autophagy contributes to the degradation of GFAP [4]. We hypothesized that increasing autophagy further in Alexander disease might lower levels of the protein.

**Fig 1:**
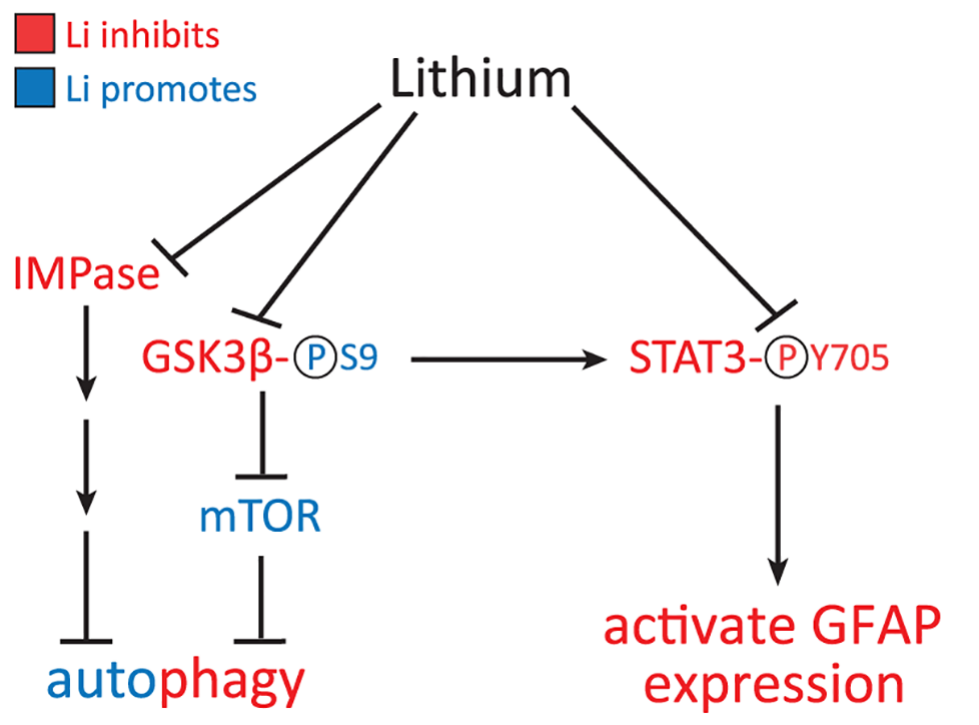
Potential pathways of lithium action on autophagy and GFAP expression. Lithium can inhibit GSK3β directly, via competition with Mg^++^, and indirectly through Akt/PKB. Inhibition of GSK3β occurs through phosphorylation (P) at Serine 9 (S9). Disinhibition of mTOR can then lead to a decrease in autophagy. Lithium can also increase autophagy through the IMPase pathway. Alternatively, lithium can inhibit STAT3 directly, or indirectly through GSK3β, by decreasing its phosphorylation at tyrosine 705 (Y705). STAT3 is then prevented from translocating to the nucleus where it would normally bind to the GFAP promoter and activate transcription. Red text indicates inhibition and blue text indicates activation of pathways by lithium.

Lithium is an autophagy-inducing drug that is commonly used as a mood stabilizer for the treatment of bipolar disorder and major depression. In cell culture models of Huntington’s disease and Parkinson’s disease, lithium increases autophagy through the inhibition of inositol monophosphatase 1 (IMPase) [15], though it has also been reported to decrease autophagy through inhibition of GSK-3β and activation of mTOR [18] (Fig 1). Lithium also affects a variety of other pathways and is neuroprotective in several models of neurodegenerative diseases including Alzheimer’s disease, Huntington’s disease, Parkinson’s disease, amyotrophic lateral sclerosis (ALS), and tauopathy (reviewed in [19]). Some protective effects associated with lithium include increased anti-apoptotic proteins, increased hippocampal neurogenesis, increased mitochondrial energetic function, reduced oxidative stress, stimulation of neurotrophic factors, and a decreased inflammatory response (reviewed in [19]). Lithium also decreases the activity of STAT3 (signal transducer and activator of transcription 3) [20–22], a transcription factor that regulates astrogliosis and promotes GFAP transcription [23] (Fig 1). Previous studies have shown that lithium can reduce activated STAT3 and decrease GFAP in rodent models of ALS, taxol-induced neuropathic pain, and lipopolysaccharide (LPS)-induced inflammation [21, 24, 25].

Due to its reported effects on autophagy, STAT3, and GFAP, we sought to test whether lithium could decrease GFAP levels in a mouse model of Alexander disease. *Gfap*-R236H knock-in mice have a mutation homologous to the common R239H mutation in humans and replicate several features of the disease, including elevated GFAP, Rosenthal fibers, an increased stress response, and an increased susceptibility to seizures [26]. We found that lithium treatment decreased GFAP protein and transcripts in several brain regions and spinal cord of *Gfap* mutant mice, though with side effects and fatalities that were dose-dependent. Markers for autophagy were unchanged, although there was diminished activation of STAT3, indicating that lithium may decrease GFAP levels through transcriptional regulation rather than protein degradation pathways.

## Materials and Methods

### Ethics Statement

All studies were carried out in accordance with the recommendations of the National Institutes of Health Guide for the Care and Use of Lab Animals and were approved by the Graduate School Animal Care and Use Committee at the University of Wisconsin-Madison (protocols G00199, G00321, and G00549).

### Mice

Knock-in mice with the *Gfap*-R236H point mutation were generated as previously described [26] and maintained as heterozygotes in the FVB/N background as previously described [27]. *Gfap-luc* transgenic mice express firefly luciferase under the control of 12 kb of the mouse *Gfap* promoter [28] and were also maintained in the FVB/N background. Mice were housed on a 10 h / 14 h dark / light cycle with 2–5 mice per cage. Male and female R236H/+ mice and +/+ littermates were used for experiments and data from each sex were analyzed separately unless otherwise indicated. Attempts were made to blind researchers to drug treatment, but due to increased urination and other mild side effects caused by LiCl, it became obvious which mice were receiving the LiCl and further efforts were not made to continue “blinding” the studies. Mice were treated in ascending order of identification number, with younger mice having higher identification numbers (newest litters were treated last). All sample processing and initial data analysis were done without knowledge of genotype or drug treatment. During all studies, mice were monitored at least once daily. Mice that were found to be inactive and unable to easily access food or water were euthanized.

### Lithium treatment via intraperitoneal injection

After genotyping, male and female mice were randomly assigned to the lithium or vehicle treatment groups. A single cage could contain mice from any or all of the 4 experimental groups (+/+ vehicle, +/+ LiCl, R236H/+ vehicle, and R236H/+ LiCl). Beginning at 6 wks of age, mice were injected intraperitoneally (i.p.) at approximately the same time each day for 30 d with a 0.6 M LiCl solution (Sigma, St. Louis, MO, USA) diluted in phosphate buffered saline (PBS) at a dose of 6 mmol/kg (10 μL per 10 g mouse) based on a protocol from Noble et al. [16]. Vehicle control mice received injections of PBS. Mice were weighed daily at the time of injection. In addition to the normal water bottle, cages contained an extra bottle of 450 mM NaCl to help prevent electrolyte imbalances that might occur with lithium treatment.

### Lithium treatment via 0.3% LiCl food pellets

Female mice were randomly assigned to the lithium or control diet treatment groups and housed in 4 large cages (2 for lithium, 2 for control diet) with up to 10 mice of both genotypes in each cage. Beginning at 6 wks of age, mice were fed rodent pellets (LabDiet 5015) containing 0.3% LiCl, based on a protocol from Tajes et al. [29]. LiCl was added to pellets by Teklad Diets, Harlan Laboratories, Inc. Control diet mice received LabDiet 5015 pellets without LiCl. Control diet and lithium mice were provided with an extra bottle of 450 mM NaCl during the treatment.

### Lithium treatment via 0.5% or 0.7% LiCl food pellets

Whole cages of 2–5 mice were randomly assigned to the lithium or control diet treatment groups [30, 31]. A single cage could contain mice of one or both genotypes (+/+ and R236H/+). Beginning at 6 wks of age, mice were fed pellets (LabDiet 5015) containing 0.2% LiCl for 1 wk, followed by 0.5% LiCl for 3 wks (4 wks total LiCl treatment) or 7 wks (8 wks total LiCl treatment) based on a protocol from Bersudsky et al. [32]. For 0.7% LiCl treatment, 6 wk old mice were fed pellets containing 0.2% LiCl for 1 wk followed by 0.7% LiCl for 3 wks (4 wks total LiCl treatment). Control diet mice received LabDiet 5015 pellets without LiCl for the whole treatment period. Control diet and lithium mice were provided with an extra bottle of 450 mM NaCl during the treatments. Mice were weighed weekly.

### Blood collection and lithium measurement

Mice were anesthetized with isoflurane and blood collected from axillary vessels. Mice were decapitated and brain collected as described below. Blood was allowed to clot for 2 h at room temperature, centrifuged at 2,000 x *g* for 10 min, and serum (supernatant) was stored at -80°C. After blood was collected from all mice in an experiment, 3–4 cages were randomly chosen per experimental group, and at least one mouse serum sample from each of those cages was randomly selected for analysis. If more than one mouse sample was used per cage, those values were averaged first before averaging with other samples. Li concentration was measured using flame atomic absorption spectrometry at the Wisconsin State Laboratory of Hygiene.

### Tissue collection

Mice were anesthetized and decapitated (as described above) or sacrificed by CO_2_. Brains were removed and dissected in ice-cold PBS into hemispheres and then regions, so that regional samples are from one half of each brain. The specific regions collected included olfactory bulb, corpus callosum, hippocampus, cerebral cortex overlying the hippocampus and underlying white matter (abbreviated as ‘cortex’), cerebellum, brain stem, and cervical spinal cord (including segments 1–3). These regions were selected based on previous studies in mouse showing locations of prominent pathology (olfactory bulb, hippocampus, and corpus callosum) [26] and based on areas of common involvement in human Alexander disease cases (white matter, brain stem, and cervical spinal cord) [3]. Tissues were immediately frozen in liquid nitrogen and stored at -80˚C until further analysis.

### Quantitative PCR

Quantitative PCR (qPCR) was performed using tissues from male mice treated with 0.5% LiCl in food for 4 wks. For each experimental group, 4–5 cages were randomly selected, then tissues from one randomly chosen mouse from each of those cages were used for qPCR. Brain regions were homogenized in 500 μL Trizol (Invitrogen/Life Technologies) using a Geno/Grinder (OPS Diagnostics LLC) at 1750 rpm for 3 min. Total RNA was isolated and purified using Trizol according to the manufacturer’s instructions. RNA concentration was quantified on a NanoDrop spectrophotometer (Thermo Fisher Scientific, Inc.) and converted to cDNA using Superscript III reverse transcriptase as in the manufacturer’s instructions (Invitrogen/Life Technologies). Primers were designed and standards generated as previously described [7] and as follows: *Gfap* (Accession # NM_010277)–forward (F)—GAC TGT GGA GAT GCG GGA TGG TGA, reverse (R)—GTG CTG GTG TGG GTG GGA ACT GAG; *Nqo1* (Accession # NM_008706)–F—CGG TAT TAC GAT CCT CCC TCA ACA, R—AGC CTC TAC AGC AGC CTC CTT CAT; 18S ribosomal RNA (Accession # NR_003278)–F—CGC CGC TAG AGG TGA AAT TCT, R—CGA ACC TCC GAC TTT CGT TCT; TATA box binding protein (*Tbp*, Accession # NM_013684)–F—GCA CAG GAG CCA AGA GTG A, R—CCC ACC ATG TTC TGG ATC TT; β-actin (*Actb*, Accession # NM_007393.3)–F—GCT GGT CGT CGA CAA CGG CTC, R—CAA ACA TGA TCT GGG TCA TCT TTT C. Reactions were performed in triplicate for standards and duplicate for samples with Power SYBR Green PCR Master Mix (Applied Biosystems/Life Technologies) on an Applied Biosystems 7500 Real-Time PCR System. Relative concentrations were calculated based on standard curves and normalized to at least two of the following: 18S ribosomal RNA, *Tbp*, or β-actin. Similar results were obtained with normalization to 18S, *Tbp*, and β-actin, therefore one set of representative data is presented in the Results and the reference gene used is indicated in the Figure legends.

### GFAP ELISA

ELISAs were performed to quantify GFAP in tissues as previously described [27]. Briefly, tissues were homogenized in 3.5 mL (brain hemisphere), 0.4 mL (olfactory bulb, corpus callosum, hippocampus, cortex, cervical spinal cord), or 0.8 mL (brain stem, cerebellum) 2% sodium dodecyl sulfate (SDS), 50 mM Tris (pH 7.4), 5 mM EDTA (pH 7.4), 1X cOmplete Protease Inhibitor Cocktail (Roche Diagnostics Corp.) and 1 mM Pefabloc (Sigma) using a Geno/Grinder, then boiled for 15 min. Total protein concentration was measured using the Pierce BCA Protein Assay kit (Thermo Fisher Scientific). For tissues, samples and GFAP standards were diluted in PBS with 0.5% Triton-X and 1% BSA. For sandwich ELISA, plates were coated with SMI-26 (1:1000, anti-GFAP monoclonal cocktail, Covance), blocked with 5% non-fat dry milk, incubated with samples and standards, then incubated with polyclonal rabbit anti-cow GFAP (1:5000; Z0334, Dako), followed by goat anti-rabbit-HRP (1:10,000), then SuperSignal ELISA Femto Maximum Sensitivity Substrate (Thermo Fisher Scientific). Chemiluminescence was measured on a GloRunner microplate luminometer (Turner Biosystems).

### Luciferase assay for *Gfap* promoter activity

Luciferase assays were performed using tissues from *Gfap-luc*-positive R236H/+ and +/+ mice using the ONE-Glo Luciferase Assay System (Promega Corporation) according to the manufacturer’s instructions. Briefly, tissues were homogenized in 300 μL (olfactory bulb, corpus callosum, hippocampus, cortex, cervical spinal cord) or 500 μL (brain stem, cerebellum) of Glo-Lysis buffer for 4 min at 1750 rpm using a Geno/Grinder and centrifuged at 1,370 x *g* for 20 min at 4°C. 40 μL of supernatant was mixed with 40 μL of luciferase substrate, incubated for 3 min, and luminescence was measured on a GloRunner microplate luminometer. Luminescence was normalized to total protein concentration measured using the Pierce BCA Protein Assay kit.

### Immunoblots

Total protein lysates used for ELISAs (in 2% SDS lysis buffer) were also used for immunoblotting. Samples were diluted in Laemmli buffer, boiled for 15 min, equal amounts of protein loaded onto 10% or 4–20% Criterion TGX precast gels (Bio-Rad), and transferred onto Immobilon-FL membranes (EMD Millipore Corporation). Membranes were blocked in SEA BLOCK (Thermo Scientific) and incubated with the following primary antibodies: rabbit anti-LC3 (1:1000 with 4–20% gel; NB100-2331, Novus Biologicals LLC), mouse anti-P62 (1:2000; 2C11, Abnova), mouse anti-GAPDH (1:10,000; 10R-G109A, Fitzgerald Industries International), rabbit anti-GAPDH (1:2500; ab9485, Abcam), rabbit anti-phospho-GSK-3α/β (Ser21/9) (1:1000; 9331, Cell Signaling Technology), and rabbit anti-STAT3-pY705 (1:2000; 9145, Cell Signaling Technology). Membranes were incubated with goat anti-rabbit Dialyte 800 (1:10,000; 35571, Thermo Scientific) and goat anti-mouse Alexa 680 (1:10,000; A21057, Invitrogen) and scanned on the Odyssey (LI-COR Biosciences). Analysis of blots was performed using Image Studio (LI-COR). LC3, p62, p70S6K, phospho-p70S6K, and phospho-GSK-3α/β were also detected with the following protocol. Membranes were blocked in 10% non-fat milk in PBS-Triton X-100, then incubated with one of the following antibodies: mouse anti-LC3 (1:400; 5H3, Nanotools), p62 (1:1000; P0067, Sigma), p70S6K (1:1000; 9202, Cell Signaling), phospho-p70S6K (1:1000; 9205, Cell Signaling), and rabbit anti-phospho-GSK-3α/β (Ser21/9) (1:1000, 9331, Cell Signaling). Membranes were incubated with anti-mouse or anti-rabbit secondary antibodies conjugated with horseradish peroxidase (1:20,000; Sigma) and developed using ECL solution (GE Healthcare).

### Statistical analysis

For body weight, ELISA, and *Gfap* promoter activity in the 0.5% and 0.7% LiCl experiments, cages were considered the unit of analysis, so that mice of the same genotype within a cage were averaged to yield a single data point [30, 31]. Results from several cages were then averaged to yield means and standard errors (SEM). Numbers of mice and cages are specified in the Results section. Comparisons among the four experimental groups were done using one-way ANOVA with post Bonferroni t-tests for 4 selected comparisons (+/+ control diet vs. +/+ LiCl, R236H/+ control diet vs. R236H/+ LiCl, +/+ control diet vs. R236H/+ control diet, and +/+ control diet vs. R236H/+ LiCl). Due to space limitations, only samples from a single brain region could be run on each ELISA, luciferase assay, or qPCR 96-well plate. Since some variability occurs between plates, statistical comparisons were not made between brain regions. For body weight analysis, statistical comparisons were made only at the final time point in each experiment. The significance level was defined as 0.05 and *P < 0.05, **P < 0.01, ***P < 0.001, ****P < 0.0001 in all figures. Statistical analyses were performed using GraphPad Prism 5.01 (GraphPad Software).

## Results

### 0.5% LiCl in food pellets for 4 wks decreases GFAP in surviving R236H/+ mice

**Fig 2:**
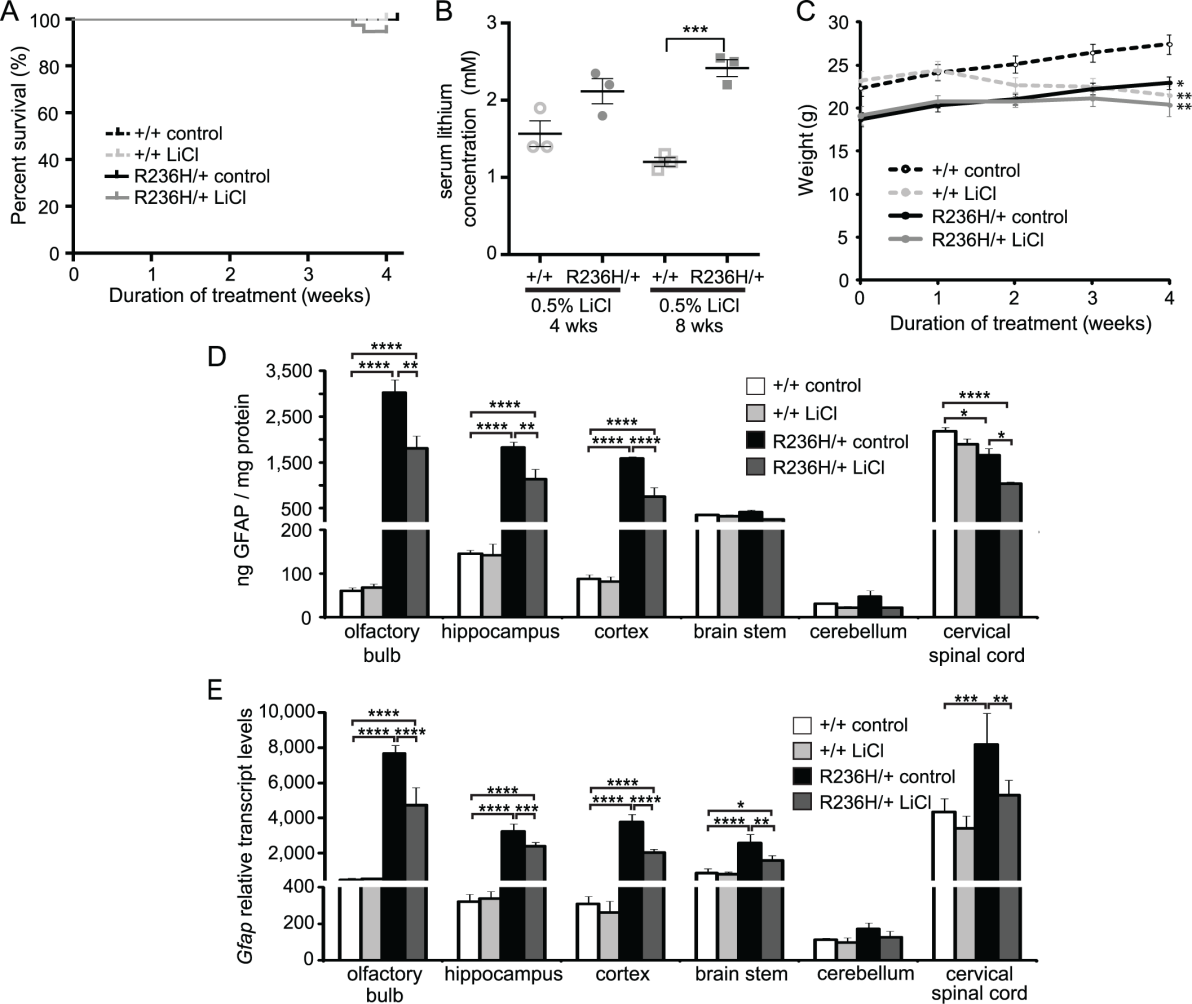
LiCl administered via 0.5% LiCl food pellets for 4 wks decreases GFAP levels in *Gfap*-R236H/+ mice. (A) Survival analysis for mice treated with 0.5% LiCl in food pellets for 4 wks. 94.7% (36/38) R236H/+ mice survived LiCl treatment while 100% of mice in other groups survived. (B) Concentrations of lithium in serum at the end of 4 or 8 wk treatments (N = 3 cages, 4 mice per genotype for 4 wks and N = 3 cages, 3–4 mice per genotype for 8 wks). (C) LiCl-treated +/+ and R236H/+ male mice had lower body weights compared with control-treated mice (N = 3–5 cages, 5–9 mice per group). (D) 0.5% LiCl treatment decreased GFAP in olfactory bulb, hippocampus, parietal cortex including underlying white matter (cortex) and cervical spinal cord of R236H/+ male mice as measured by ELISA (N = 3–5 cages, 5–9 mice per group). (E) 0.5% LiCl treatment decreased *Gfap* transcript levels relative to *Tbp* in all regions except cerebellum of R236H/+ male mice (N = 4–5 mice per group). Error bars are SEM in B-D and SD in E. ****P < 0.0001, ***P < 0.001, **P < 0.01, *P < 0.05. * is versus +/+ control in C.

To test whether lithium can improve the phenotype of a mouse model of Alexander disease, we treated mice with lithium using several different methods. In preliminary experiments, we employed daily i.p. injections for 30 d (S1 Fig) or 0.3% LiCl in food pellets for 30 d (S2 Fig), but neither decreased GFAP protein in more than one of the six brain regions tested. We then increased the dose and fed mice food pellets containing 0.5% LiCl. Beginning at 6 wks of age, male and female mice were fed 0.2% LiCl food for 1 wk, followed by 0.5% LiCl food for 3 wks. 94.7% (36/38) of the R236H/+ mice fed LiCl survived the 4 wks of treatment, while all of the +/+ control diet (22/22), +/+ LiCl (22/22), and R236H/+ control diet (37/37) mice survived (Fig 2A). Mice treated with this dose of LiCl showed mild side effects, including polyuria and occasional jitteriness. The two mice that died during treatment (1 male, 1 female) had no additional signs of illness before being found dead. Lithium concentrations in the serum were measured in a subset of surviving mice on the last day of treatment and found to be 1.6 ± 0.3 mM in +/+ mice, and 2.1 ± 0.3 mM in R236H/+ mice (Fig 2B). Mice fed the control diet had undetectable levels of lithium (N = 3 cages, 3 mice; < 0.1 mM).

R236H/+ mice typically weigh less than +/+ littermates, and we next tested whether LiCl treatment improved the body weight phenotype. However, R236H/+ LiCl mice maintained a steady weight during the course of treatment and did not gain weight like control-fed littermates (Fig 2C). Wild type mice fed the LiCl diet experienced mild weight loss during the 4 wks of treatment (Fig 2C).

We next tested the effect of 0.5% LiCl treatment on GFAP levels in the mice. Using a GFAP ELISA, we found that the 0.5% LiCl food treatment for 4 wks decreased GFAP protein levels in several brain regions of male and female R236H/+ mice, with minor variations between the sexes. In male R236H/+ mice, LiCl decreased GFAP protein in olfactory bulb, hippocampus, cortex, and cervical spinal cord (Fig 2D), while in female R236H/+ mice, LiCl decreased GFAP in hippocampus, cortex, brain stem, cerebellum, and cervical spinal cord (S3 Fig). GFAP protein did not change in any regions of +/+ mice treated with 0.5% LiCl.

We next analyzed GFAP expression at the level of mRNA. *Gfap* transcript levels changed in a pattern similar to protein levels. In male R236H/+ mice, LiCl decreased *Gfap* transcripts in olfactory bulb, hippocampus, cortex, brain stem, and cervical spinal cord, while LiCl did not change *Gfap* transcript levels in +/+ mice (Fig 2E).

### 0.7% LiCl in food pellets for 4 wks does not further decrease GFAP

We next tested whether a higher dose of 0.7% LiCl would have a greater effect, but this dose in food pellets for 4 wks did not further decrease GFAP compared with the 0.5% LiCl treatment. LiCl at 0.7% decreased GFAP only in cerebellum and cervical spinal cord of male R236H/+ mice, and in olfactory bulb and cerebellum of female R236H/+ mice (S4 Fig). 93.8% (15/16) of the R236H/+ mice fed LiCl survived the 4 wks of treatment, while all of the +/+ control diet (13/13), +/+ LiCl (14/14), and R236H/+ control diet (18/18) mice survived. Lithium-treated mice showed mild side effects, including polyuria and occasional jitteriness. Serum lithium levels in a subset of surviving mice were 1.3 ± 1.0 mM in +/+ mice and 3.0 ± 0.7 mM in R236H/+ mice.

### 0.5% LiCl in food pellets for 8 wks decreases GFAP in surviving R236H/+ mice

**Fig 3:**
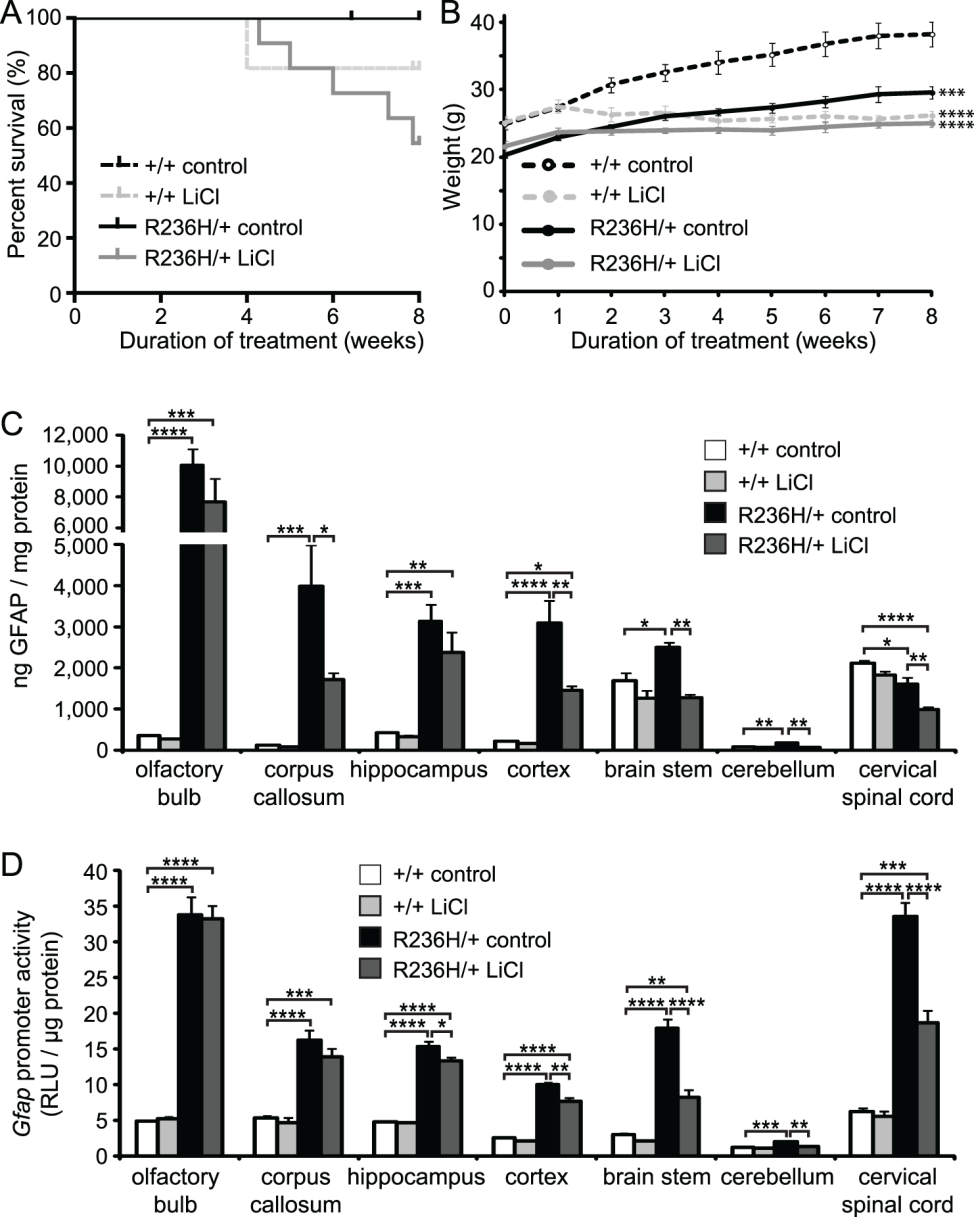
LiCl administered via 0.5% LiCl food pellets for 8 wks decreases GFAP levels in surviving *Gfap*-R236H/+ mice. (A) 81.8% (9/11) +/+ mice and 54.5% (6/11) R236H/+ mice survived 8 wks of LiCl treatment. All mice survived control diet treatment. (B) LiCl-treated +/+ and R236H/+ male mice had lower body weights compared with control-treated mice (N = 4–6 cages, 6–11 mice per group). (C) 0.5% LiCl treatment decreased GFAP in corpus callosum, parietal cortex including underlying white matter (cortex), brain stem, cerebellum, and cervical spinal cord of R236H/+ male mice as measured by ELISA (N = 3–4 cages, 4–6 mice per group). (D) 0.5% LiCl decreased *Gfap* promoter activity in hippocampus, cortex, brain stem, cerebellum, and cervical spinal cord of R236H/+; *Gfap*-*luc* mice (N = 3–5 cages, 4–5 mice per group). Error bars are SEM. ****P < 0.0001, ***P < 0.001, **P < 0.01, *P < 0.05. * is versus +/+ control in B.

We next tested whether lengthening the duration of 0.5% LiCl treatment would further decrease GFAP levels in R236H/+ mice. Beginning at 6 wks of age, male mice were fed 0.2% LiCl for 1 wk, followed by 0.5% LiCl for 7 wks. For the 8 wk treatment, R236H/+ mice were crossed with *Gfap*-*luc* transgenic mice that express luciferase under control of the mouse *Gfap* promoter, in order to test the effects of lithium on *Gfap* promoter activity. Since mRNA levels can also be regulated by degradation mechanisms, GFAP promoter activity can provide an additional, more direct measure of GFAP transcriptional activity. The 8 wk treatment resulted in a higher mortality rate among +/+ and R236H/+ mice receiving the 0.5% LiCl food, compared with the 4 wk treatment. All of the +/+ control diet (10/10) and R236H/+ control diet (6/6) mice survived until the end of the treatment, but only 81.8% (9/11) of +/+ LiCl and 54.5% (6/11) of R236H/+ LiCl mice survived 8 wks of treatment (Fig 3A). The seven mice that did not survive died at or after 4 wks of treatment. Two of the mice (one +/+, one R236H/+) were lethargic with some possible tremors and after 1–2 h of observation and unsuccessful attempts to help them eat and drink, the mice were euthanized. The remaining five mice (one +/+, four R236H/+) were jittery on occasion, but displayed no other signs of illness before being found dead. Serum lithium levels were higher in R236H/+ mice (2.4 ± 0.2 mM) compared with +/+ mice (1.2 ± 0.1 mM; Fig 2B). Mice fed the control diet had undetectable levels of lithium (N = 3 cages, 3 mice; < 0.1 mM). Similar to the 4 wk treatment, R236H/+ mice maintained a steady weight throughout the treatment, neither gaining nor losing significant body weight (body weight analysis includes all available data from mice euthanized or found dead; Fig 3B). +/+ mice had mild weight loss through the 4 wk time point, then maintained a steady weight until 8 wks (Fig 3B).

We next quantified GFAP protein levels and found that 0.5% LiCl treatment for 8 wks decreased GFAP in corpus callosum, cortex, brain stem, cerebellum, and cervical spinal cord of surviving R236H/+ mice (Fig 3C). Using a cross to a *Gfap-*luciferase reporter mouse to provide an indirect measure of promoter activity (previously shown to be increased in the R236H/+ mice [8]), we found that *Gfap* promoter activity was also decreased in hippocampus, cortex, brain stem, cerebellum, and cervical spinal cord of the GFAP mutants that survived lithium treatment (Fig 3D).

### 0.5% LiCl in food pellets for 4 wks decreases the antioxidant stress response

**Fig 4:**
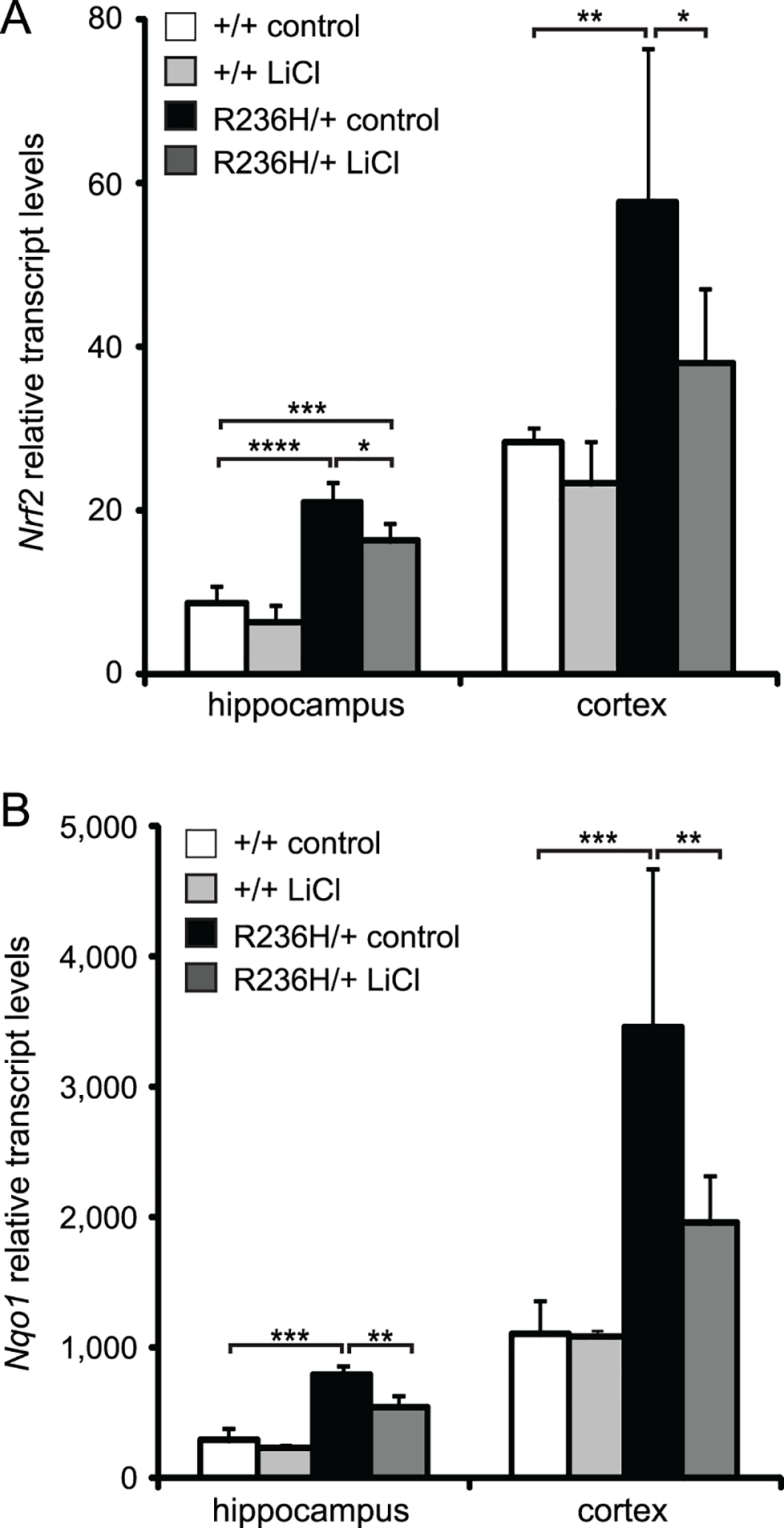
LiCl administered via 0.5% LiCl food pellets for 4 wks decreases the antioxidant stress response. (A) LiCl decreases *Nrf2* transcripts in hippocampus and cortex of R236H/+ mice (N = 5 male mice per group). (B) LiCl decreases *Nqo1* transcript levels in hippocampus and cortex of R236H/+ mice (N = 4–5 male mice per group). Error bars are SD. Data are normalized to 18S. ****P < 0.0001, ***P < 0.001, **P < 0.01, *P < 0.05.

R236H/+ mice have an elevated antioxidant stress response, implying oxidative stress, that increases with age [26], and we next wanted to test whether LiCl treatment would decrease this stress response. Nrf2 (Nfe2l2, nuclear factor, erythroid derived 2, like 2) is a transcription factor that regulates many genes involved in the protective antioxidant stress response and is elevated in R236H/+ mice [26]. We examined cortex and hippocampus of male mice and found that 0.5% LiCl food decreased *Nrf2* transcripts in the R236H/+ mice (Fig 4A). NAD(P)H dehydrogenase, quinone 1 (*Nqo1)* is an antioxidant enzyme that is regulated by Nrf2 and transcripts of *Nqo1* are elevated in R236H/+ mice [27]. 0.5% LiCl decreased *Nqo1* transcript levels in cortex and hippocampus of R236H/+ mice (Fig 4B). These results indicate that the antioxidant stress response is decreasing in regions where lithium decreases GFAP.

### 0.5% LiCl in food pellets for 4 wks does not increase autophagy in R236H/+ mice

**Fig 5:**
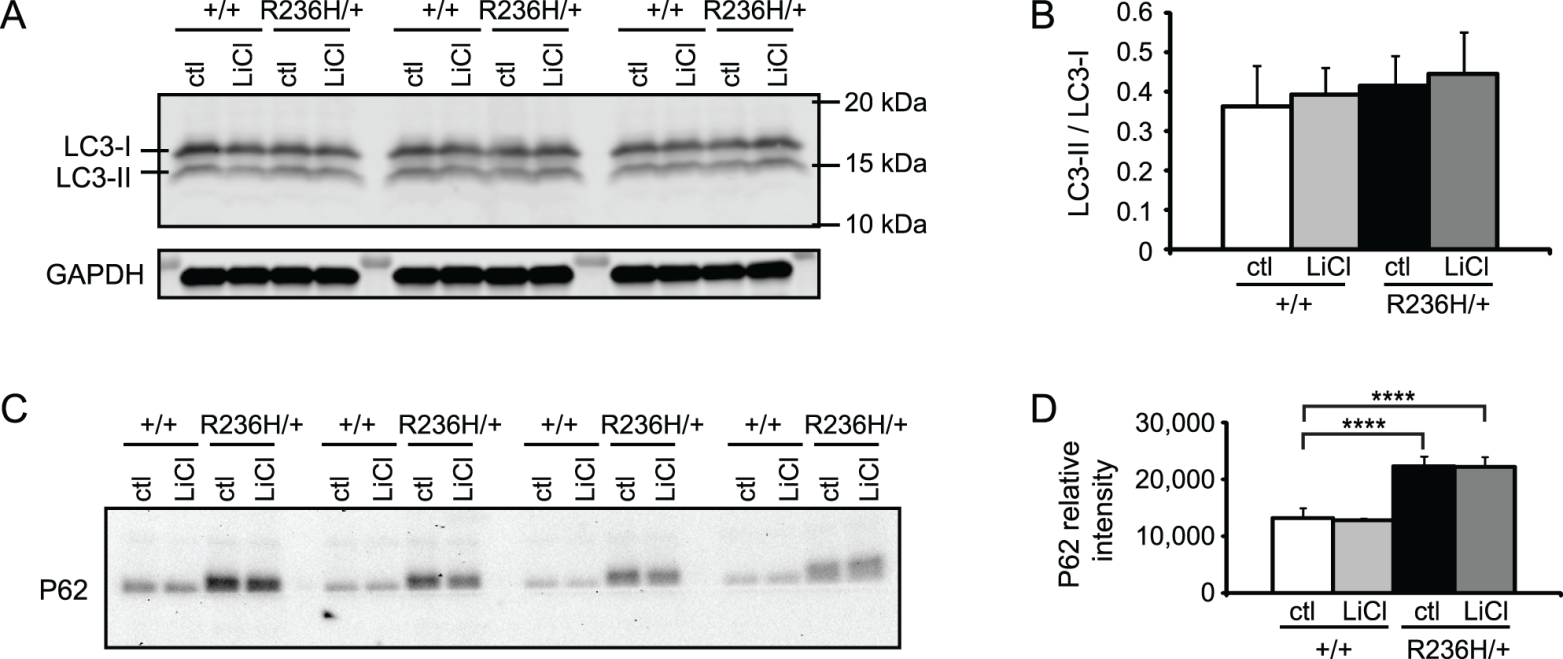
LiCl administered via 0.5% LiCl food pellets for 4 wks does not increase markers for autophagy in *Gfap-*R236H/+ mice. Each lane of the immunoblots is tissue from one mouse. Immunoblots for LC3-I and LC3-II in (A) did not detect a change in parietal cortex (and underlying white matter) with LiCl treatment. LC3-II bands normalized to LC3-I are quantified in B (N = 3–4 mice from 3–4 cages per genotype, and is representative of 3 blots). LC3-II normalized to GAPDH gave similar results and is not shown. P62 was increased in control diet R236H/+ mouse olfactory bulb compared with control diet +/+, but LiCl did not change P62 levels in GFAP+/+ or R236H/+ mice (C-D). P62 was normalized to total protein loaded. Error bars are SEM. ****P < 0.0001.

Previous studies have shown that lithium can increase autophagy through the IMPase pathway [15], and increasing autophagy with rapamycin decreases GFAP levels in astrocytoma cells expressing Alexander disease mutant GFAP [4]. Although there is some evidence that autophagy is spontaneously elevated in Alexander disease [4], we considered whether the lithium-induced decrease in GFAP might be associated with a further increase in this degradation pathway. LC3-II is found in phagophore and autophagosome membranes and is commonly used as an indicator of autophagosome number [33] (reviewed in [34]). Immunoblotting showed LC3-II and the LC3-II / LC3-I ratio were not different between control diet +/+ and control diet R236H/+ mice. In addition, 0.5% LiCl for 4 wks did not change LC3-II levels or LC3-II / LC3-I ratios in female R236H/+ cortex, a region where LiCl decreased GFAP levels (Fig 5A and 5B). To confirm that LiCl was decreasing GFAP in the absence of an increase in autophagy, we also examined p70S6K, a substrate of mTOR involved in autophagy (reviewed in [35]). Considerable variability in phosphorylated p70S6K (phospho-p70S6K) and total p70S6K between mice resulted in no overt changes with lithium treatment (S5A and S5B Fig). P62 is a substrate of autophagy that also accumulates in Rosenthal fibers [36]. We found that P62 was increased in control diet R236H/+ mouse olfactory bulb compared with control diet +/+, but lithium treatment did not change P62 levels (Fig 5C and 5D). Lithium treatment also did not change P62 levels in cortex (S5E and S5F Fig). Together, these results suggest that 0.5% LiCl for 4 wks does not increase autophagy.

Lithium is thought to exert some of its effects on brain through the GSK3β pathway. Lithium both directly inhibits GSK3β and also indirectly inhibits GSK3β through phosphorylation at serine 9 (pS9-GSK3β), a pathway through which it may inhibit autophagy and promote STAT3 activity [20]. We found that 0.5% LiCl for 4 wks did not consistently increase pS9-GSK3β in cortex (data not shown) or hippocampus (S5C and S5D Fig). This indicates that the effects of lithium on GFAP levels may not involve GSK3β.

### 0.5% LiCl in food pellets for 4 wks inhibits STAT3

**Fig 6:**
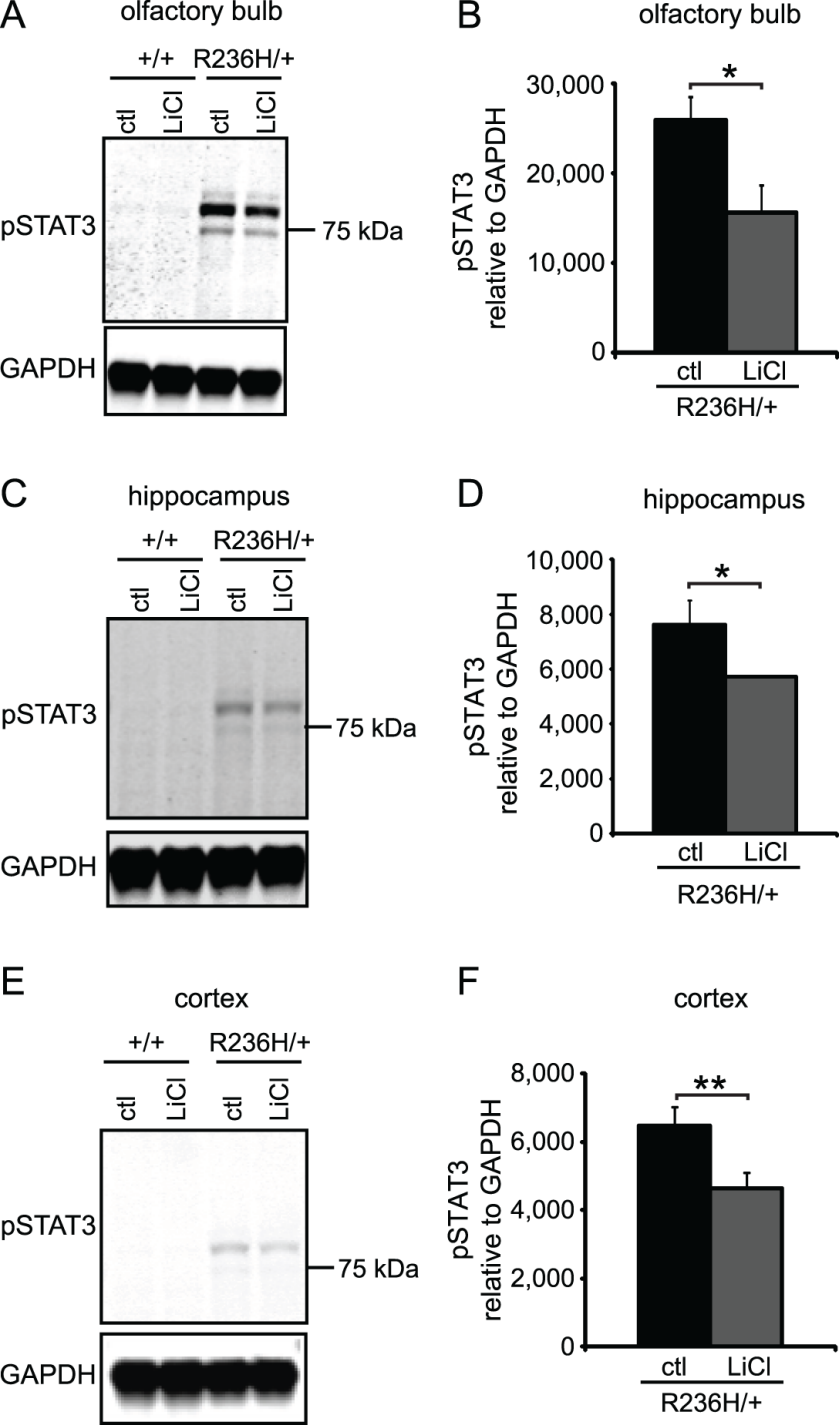
LiCl administered via 0.5% LiCl food pellets for 4 wks decreases pY705-STAT3 (pSTAT3) levels in GFAP-R236H/+ mice. Representative immunoblots for pSTAT3 in olfactory bulb (A), hippocampus (C), and parietal cortex including underlying white matter (E). pSTAT3 bands normalized to GAPDH are quantified in B, D, and E (N = 3–4 mice from 3–4 cages per group; quantification is of main dark band; uncropped blots are in S6 Fig). Error bars are SD. **P < 0.01, *P < 0.05.

Another pathway through which lithium may be decreasing GFAP is the Janus kinase (JAK)- signal transducer and activator of transcription (STAT) pathway. STAT3 is a transcription factor involved in astrogliogenesis and regulation of GFAP [23] and reactive astrogliosis [37]. Phosphorylation of STAT3 at Tyr-705 (pY705-STAT3) by the JAK family of kinases is required for its activation and translocation to the nucleus (reviewed in [38]). Lithium has been shown to inhibit STAT3 and suppress astrogliogenesis in culture by decreasing pY705-STAT3 [22]. Using immunoblotting, we found that pY705-STAT3 was undetectable in +/+ control diet mice and increased in R236H/+ control diet mice (Fig 6), indicating that the R236H mutation increases activation of STAT3. In R236H/+ mice, 0.5% LiCl treatment for 4 wks caused a small but consistent decrease in pY705-STAT3 in all 3 brain regions examined: olfactory bulb, hippocampus, and cortex (Fig 6). These results suggest that lithium may be decreasing GFAP in R236H/+ mice through effects on STAT3 phosphorylation.

## Discussion

Alexander disease is a fatal neurodegenerative disorder for which only supportive treatments exist. For such a rare disorder (incidence less than 1 in a million based on one study [39]), identifying potential therapeutics from drugs that are already approved by the FDA is a promising approach. Lithium has a long history of application in the treatment of bipolar disorder, and in experimental studies is protective in several models of neurodegenerative diseases. Based on previous reports that lithium increases the autophagy pathway for protein degradation [15, 18], and other studies where lithium treatment was associated with a decrease in GFAP itself [21, 24, 25], we tested whether lithium would decrease GFAP accumulation in a mouse model of Alexander disease. We show here that lithium treatment decreases GFAP levels in several brain regions of R236H/+ mice, but likely through transcriptional mechanisms, and with a narrow therapeutic range.

### Molecular mechanisms of lithium action

Previous studies have indicated that mutant GFAP can be degraded through autophagic mechanisms, possibly involving reduction in mTOR activity [4]. In a previous study, LC3-II, commonly used as an indicator of autophagosome number, was increased in astrocytes cultured from GFAP-R236H/+ mice, autophagic vacuoles were detected near Rosenthal fibers in R236H/+ mouse brain, and LC3-II was increased and phospho-mTOR and phospho-p70S6K were both decreased in human astrocytoma cell lines expressing mutant GFAP [4]. In contrast, we did not detect changes in LC3-II, the LC3-II / LC3-I ratio, phospho-p70S6K, or total p70S6K in R236H/+ mice compared with wild-type mice. Tang et al. [4] also did not detect a change in total p70S6K levels in mutant GFAP expressing cells, though they did detect a decrease in phospho-p70S6K. Studies in mouse brain could be complicated by the contributions of other cell types that mask specific effects in astrocytes.

Previous studies suggest that lithium can increase autophagy through the IMPase pathway [15], but we did not detect changes either in autophagosome markers (such as LC3-II) or mTOR pathway proteins (such as p70S6K) in lithium-treated mice. It remains possible that lithium increased autophagic flux while autophagosome number (and LC3-II) remained stable. Autophagy is also known to be induced via the mTOR pathway, previously shown to be activated in cell lines expressing mutant GFAP [4]. However we observed a high degree of variability, and consequently no significant change, in the levels of phospho-p70S6K and p70S6K with lithium treatment, indicating autophagy was not affected via the mTOR pathway. However, p70S6K activity would not be expected to change if lithium acts through an mTOR-independent pathway, a possibility suggested by previous studies [15].

GFAP is also regulated by the proteasome system [9, 10] and it is possible lithium could decrease GFAP levels by increasing proteasome activity or by increasing the efficiency of GFAP degradation by the proteasome. To our knowledge, there is no evidence for a direct effect of lithium on the proteasome, but increased proteasomal degradation of GFAP could be a secondary effect of a decreased stress response and mutant GFAP [9, 10].

In addition to its potential effects on degradation of GFAP, lithium could also influence GFAP levels through transcriptional control. The transcription factor STAT3 is involved in astrocyte differentiation [23, 40] and reactive gliosis [37], and it has a known binding site in the GFAP promoter [23]. Previous studies indicate lithium may inhibit STAT3 through the serine/threonine kinase GSK3 [20, 21] (Fig 1). GSK3 is normally active and becomes inactivated when phosphorylated, for example, upon lithium treatment [41, 42]. Previous studies showed increased autophagy and inhibition of GSK3β in lithium treated mice [16, 24], but in our experiments, lithium did not consistently increase phosphorylated GSK3β. Our inability to replicate the effect of lithium on GSK3β may be related to mouse strain-dependent effects of lithium [43]. Nevertheless, our results suggest that decreases in pSTAT3 and GFAP occur independently of GSK3β. Zhu et al. also found that lithium may inhibit STAT3 independently of GSK3 [22]. Lithium has many effects in the brain [44], and it remains possible that its effects on STAT3 activation are indirect, due to suppression of gliosis through other pathways.

Interestingly, lithium treatment did not decrease GFAP in +/+ mice, in whom pSTAT3 was undetectable. This suggests the effects of lithium were specific to R236H/+ mice who had elevated pSTAT3 and supports the possibility that lithium decreases GFAP through STAT3 inhibition.

As indicators of the antioxidant stress response, we measured *Nrf2* and *Nqo1* transcript levels following lithium treatment. Under conditions of stress, Nrf2 activates a program of genes meant to counteract stressful conditions and protect the cell. As the stress stimulus subsides, we would expect the helpful Nrf2 response to subside as well. We previously found that *Nrf2* and *Nqo1* are elevated in R236H/+ mice [26] and that a further increase in Nrf2 is helpful and leads to a decrease in GFAP in R236H/+ mice [27]. We also found that the Nrf2 activation was diminished in the context of protective over-expression of αB-crystallin [45]. For the current study, we used *Nrf2* and *Nqo1* as indicators of the antioxidant stress response and found that after lithium treatment, the stress response had decreased. This data also suggests that the helpful effects of lithium are not mediated through Nrf2 pathways since lithium did not increase *Nrf2* and *Nqo1* transcripts.

Ultimately the levels of GFAP in brain reflect a balance between synthesis and degradation. The half-life of GFAP in vivo has only been studied twice, once using a radioactive tracer (and reporting a half-life of ~9 weeks in mouse spinal cord) [46] and once using stable isotopic labeling for a more global measure of turnover (and reporting a half-life of 28 days in mouse brain) [47]. Whether these kinetics are applicable when some of the GFAP is mutated and present in protein aggregates is the subject of active investigation. Nevertheless, we found no evident difference in the degree of GFAP suppression when we doubled the treatment duration from 4 to 8 weeks, suggesting either that lithium had reached its maximal effect with these doses or that adaptation to the lithium takes place.

### Variability between brain regions

We found noticeable differences in the degree of GFAP suppression by lithium as a function of anatomic region. In mouse, rat, and human, baseline levels of GFAP vary considerably across regions [48, 49], which could be attributed to differences in astrocyte density and/or heterogeneity among distinct astrocyte populations. In addition, it is possible that non-uniformity of lithium distribution within the CNS following peripheral administration could contribute to variability in response [50], although in our hands the GFAP response was greater in corpus callosum than hippocampus (the opposite direction of the differences in drug concentration found by Thellier [50]).

### Toxicity of lithium treatment

Lithium is known to have a narrow therapeutic range, with plasma concentrations of 0.5–1.2 meq/L considered a safe therapeutic range in human patients [51]. Toxic effects usually occur at 1.5 meq/L and dangerous life-threatening side effects can occur at concentrations above 2.0 meq/L [51]. In our studies we focused on doses ranging from 0.3% to 0.7% in the diet, yet even with 0.5% LiCl for 4 wks, serum concentrations were at or above 1.5 meq/L (1.5 mM Li). Lithium kinetics differ between humans and mice [52], which complicates the linkages between dosing and blood levels. Nevertheless, the already broad spectrum of lithium targets appears to widen at higher concentrations [53]. It is possible cell death could account for changes in GFAP expression and this is a limitation of the present study. However, examination of limited tissues did not reveal any necrotic tissue or pyknotic nuclei in lithium-treated brains, and previous studies in rats also did not find any morphological changes or cell death following toxic levels of lithium [54, 55].

In conclusion, we show that lithium decreases GFAP in a mouse model of Alexander disease, possibly through transcriptional mechanisms involving STAT3. However, its narrow therapeutic range complicates the use of lithium for therapeutic purposes. In addition, there is little experience in using lithium in young pediatric patients (who constitute a significant portion of the Alexander disease population), although recently lithium was reported to be well tolerated in a small trial conducted for Canavan disease on 6 patients with an average age of 9.5 months [56].

## Supporting Information

S1 Fig LiCl administered daily via i.p. injection for 30 days does not have significant effects on GFAP-R236H/+ mice. (A) All mice survived the LiCl i.p. treatment. (B-C) R236H/+ mice have lower body weights compared with +/+ littermates and LiCl treatment does not change this in male (B) or female mice (C) (N = 7–10 mice per female group and 4–10 mice per male group). (D-E) LiCl i.p. treatment decreased GFAP in parietal cortex (including underlying white matter) of female R236H/+ mice (D, N = 5–6 mice per group) and in cerebellum of R236H/+ male mice (E, N = 4–5 mice per group) but did not affect GFAP levels in other brain regions. Error bars are SD. ****P < 0.0001, ***P < 0.001, **P < 0.01, *P < 0.05. * is versus +/+ control in B and C.
(EPS)

S2 Fig LiCl administered via 0.3% LiCl food pellets for 30 days does not have significant effects on female GFAP-R236H/+ mice. (A) All mice survived the 0.3% LiCl food treatment. (B) R236H/+ mice have lower body weights compared with +/+ littermates and LiCl treatment does not change this (N = 5–9 mice per group). (C) 0.3% LiCl food treatment did not affect GFAP levels in any brain regions (N = 6–9 mice per group). Error bars are SD. ***P < 0.001.
(EPS)

S3 Fig 0.5% LiCl in food for 4 wks decreases GFAP in female GFAP-R236H/+ mice. (A) +/+ LiCl and R236H/+ LiCl mice have lower body weights than control mice (N = 4–6 cages, 5–11 mice per group). (B) LiCl decreases GFAP in all regions of R236H/+ mice, except olfactory bulb (N = 4–6 cages, 5–10 mice per group). Error bars are SEM. ****P < 0.0001, ***P < 0.001, *P < 0.05. * is versus +/+ control in A.
(EPS)

S4 Fig LiCl administered via 0.7% LiCl food pellets for 4 wks decreases GFAP in only a few brain regions. (A) 93.7% (15/16) of R236H/+ mice survived 4 wks of LiCl treatment, while all +/+ mice survived LiCl treatment and all mice survived control treatment. (B-C) 0.7% LiCl treatment decreased body weights in +/+ and R236H/+ male (B) and female (C) mice (N = 4–5 cages, 5–8 mice per male group and 4–6 cages, 5–12 mice per female group). (D) 0.7% LiCl food treatment decreased GFAP in cerebellum and cervical spinal cord of male R236H/+ mice (N = 4–5 cages, 5–8 mice per group). (E) 0.7% LiCl treatment decreased GFAP in olfactory bulb and cerebellum of female R236H/+ mice (N = 4–5 cages, 6–11 mice per group). Error bars are SEM. ****P < 0.0001, ***P < 0.001, **P < 0.01, *P < 0.05. * is versus +/+ control and ‡ is R236H/+ control versus R236H/+ LiCl in B and D.
(EPS)

S5 Fig 0.5% LiCl in food for 4 wks caused variable effects on p70S6K and GSK3β. (A-B) Immunoblots of male cortex showed substantial variability in the autophagy protein p70S6K and phospho-p70S6K with LiCl treatment. All p70S6K samples were run together on the same gel and all p-p70S6K samples were run together on a second gel. Separation of the lanes by the black boxes was done to align the p70S6K samples with their corresponding p-p70S6K sample from the same mouse. (C-D) LiCl treatment did not consistently increase phosphorylation of GSK3β in GFAP+/+ and R236H/+ hippocampus. (E-F) LiCl treatment did not change P62 levels in male cortex (N = 3–5 mice from 3–5 cages per group). Error bars are SEM. *P < 0.05.
(EPS)

S6 Fig Uncropped blots. Whole uncropped blots for STAT3 and p70S6K.
(EPS)
